# The impact of transmural multiprofessional simulation-based obstetric team training on perinatal outcome and quality of care in the Netherlands

**DOI:** 10.1186/1472-6920-14-175

**Published:** 2014-08-21

**Authors:** Franyke R Banga, Sophie E M Truijens, Annemarie F Fransen, Jeanne P Dieleman, Pieter J van Runnard Heimel, Guid S Oei

**Affiliations:** 1 Department of Obstetrics and Gynaecology, Máxima Medical Centre, De Run 4600, P.O. Box 7777, Veldhoven, 5500 MB, The Netherlands; 2 MMC Academie, Máxima Medical Centre, Veldhoven, The Netherlands; 3 Department of Electrical Engineering, Eindhoven University of Technology, Eindhoven, The Netherlands

**Keywords:** Multiprofessional, Simulation-based obstetric team training, Deliberate practice, Crew resource management, Quality of care

## Abstract

**Background:**

Perinatal mortality and morbidity in the Netherlands is relatively high compared to other European countries. Our country has a unique system with an independent primary care providing care to low-risk pregnancies and a secondary/tertiary care responsible for high-risk pregnancies. About 65% of pregnant women in the Netherlands will be referred from primary to secondary care implicating multiple medical handovers. Dutch audits concluded that in the entire obstetric collaborative network process parameters could be improved. Studies have shown that obstetric team training improves perinatal outcome and that simulation-based obstetric team training implementing crew resource management (CRM) improves team performance. In addition, deliberate practice (DP) improves medical skills. The aim of this study is to analyse whether transmural multiprofessional simulation-based obstetric team training improves perinatal outcome.

**Methods/Design:**

The study will be implemented in the south-eastern part of the Netherlands with an annual delivery rate of over 9,000. In this area secondary care is provided by four hospitals. Each hospital with referring primary care practices will form a cluster (study group). Within each cluster, teams will be formed of different care providers representing the obstetric collaborative network. CRM and elements of DP will be implemented in the training. To analyse the quality of care as perceived by patients, the Pregnancy and Childbirth Questionnaire (PCQ) will be used. Furthermore, self-reported collaboration between care providers will be assessed. Team performance will be measured by the Clinical Teamwork Scale (CTS). We employ a stepped-wedge trial design with a sequential roll-out of the trainings for the different study groups.

Primary outcome will be perinatal mortality and/or admission to a NICU. Secondary outcome will be team performance, quality of care as perceived by patients, and collaboration among care providers.

**Conclusion:**

The effect of transmural multiprofessional simulation-based obstetric team training on perinatal outcome has never been studied. We hypothesise that this training will improve perinatal outcome, team performance, and quality of care as perceived by patients and care providers.

**Trial registration:**

The Netherlands National Trial Register, http://www.trialregister.nl/NTR4576, registered June 1, 2014

## Background

### Perinatal mortality in the Netherlands

Perinatal mortality and morbidity in the Netherlands is relatively high compared to other countries in Europe, shown by Peristat I (data of 1999) [1] and Peristat II (data of 2004) [2-4]. Initiated by the Dutch Minister of Health, a Committee Project group Pregnancy and Birth was started in 2008, just after publication of Peristat II. The main goal was to improve quality of obstetric care in the Netherlands. Beside several implementations such as regional Obstetric Cooperatives and the Dutch Perinatal Audit, a nation-wide research programme on pregnancy and birth of the Netherlands Organization for Health, Research and Development (ZonMw) was developed. Recently, the data of the third Euro-Perinatal European Perinatal Health Report (data of 2010) were launched [5]. Perinatal mortality in the Netherlands has declined with 14% between 2004 and 2010, however the current mortality rate still represents a poor international position, which is even more remarkable considering that the Netherlands was ranked second highest in Europe concerning welfare [6]. In 2004, the Netherlands featured the third highest perinatal mortality (out of 26 countries). In 2010, the Netherlands ranked the sixth highest perinatal mortality out of 29 European countries. The perinatal mortality in the Netherlands should decrease faster than in other European countries in order to be ranked in the top.

### Dutch system

**Figure 1:**
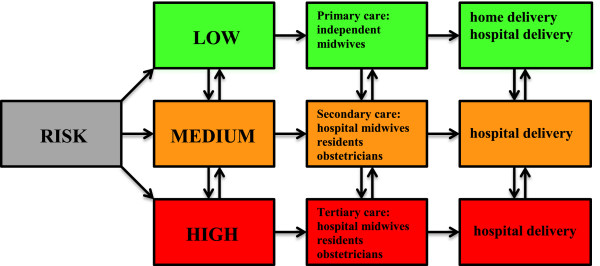
**Obstetric system in the Netherlands: based on risk selection organised in independent primary, secondary and tertiary care.** The arrows reflect possible referrals during pregnancy and delivery.

The Netherlands has an estimated population of 16.7 million. In the Netherlands, around 175,000 children are born yearly, of which around 1,500 babies die (perinatal mortality). Obstetric care is organised in low-risk primary care, medium-risk secondary, and high-risk tertiary care (Figure 1). Primary care concerning low-risk pregnancies is represented by independent midwives (and general practitioners). A low-risk pregnant woman has the possibility of planning her delivery either at home or in a primary care hospital setting, both under responsibility of her own independent midwife. The Netherlands has a high rate of home delivery, although rate is declining from 26% home deliveries in 2007 to 15.6% in 2012 [7]. Secondary care is regionally organised in 92 hospitals of which 10 hospitals provide tertiary care in a perinatology centre facilitating a Neonatal Intensive Care Unit (NICU) and an Obstetric High Care unit (OHC). Secondary and tertiary care is provided by obstetric nurses, secondary care (hospital) midwives, residents and obstetricians, working together in teams. During pregnancy and/or delivery a pregnant woman may evolve from low-risk to medium- or high-risk, followed by a referral from primary to secondary or tertiary care. Indications for referral are defined in the ‘Obstetric Indication List’ [8]. Perinatal data of 2007 show a huge shift between primary and secondary/tertiary care: the intention (42%) and reality (26%) to deliver at home, the intention (42%) and reality (16%) to deliver in primary care in hospital, and the intention (16%) and reality (61%) to deliver in secondary care in hospital [9-14]. This shift between primary and secondary care is getting more extensive when interpreting recent perinatal data of 2012: about 85% of pregnancies starts in primary care and 15% in secondary/tertiary care. Finally, 30% will give birth in primary care and 70% in secondary/tertiary care. This means that about 65% of all pregnancies will be referred from primary to secondary/tertiary care, during pregnancy or delivery [7]. This extensive shift between the different care levels results in multiple medical handovers, potentially causing errors in communication and process management.

### Risk of home delivery

A recent Dutch study showed a higher risk of delivery related perinatal mortality among women with planned delivery in primary care (at home or in hospital) compared to women who started delivery in secondary care. An even higher risk of perinatal mortality was found in women who were referred from primary to secondary care during delivery [15]. Another Dutch study did not find a significant difference between a planned home and hospital delivery among low-risk women in primary care [16]. However, the results of these two Dutch studies cannot be compared because different groups and different comparisons were studied: the first study compared planned primary care delivery with planned secondary care delivery while the second study compared planned home delivery with planned hospital delivery in primary care. The British Birthplace cohort study concluded that nulliparous low risk women with a planned home delivery have an increased incidence of adverse perinatal outcome. For multiparous women, there were no significant differences in adverse perinatal outcome by planned place of birth. Interventions during delivery were substantially lower in all non-obstetric unit settings [17].

### Causes of perinatal death

Analysis of Dutch data showed that 85.2% of perinatal mortality is caused by one or more of the four following disorders, together the so called Big 4: small for gestational age (SGA: birth weight below 10^th^ percentile), preterm delivery before 37 weeks of gestation, congenital anomaly and low Apgar score (Apgar score below 7). Big 4 disorders are overlapping each other often, creating a multiple diagnosis. Accumulation of Big 4 disorders obviously increases mortality rate. The group with exclusively one Big 4 disorder causing perinatal death is small. Of all pregnancies, 16.3% represents a Big 4 disorder. Of all Big 4 pregnancies, 29% starts delivery in primary care. This indicates that risk selection is inadequate. [9,18-20]. These data suggest that evaluation and improvement of process management of pregnancies complicated by a Big 4 disorder will be beneficial for perinatal outcome.

### Process parameters and communication audit

Analysis of all term perinatal death cases in 2010 by the Dutch Perinatal Audit revealed one or more substandard factors (SSF) in 52% of the cases. In 56% of the cases with SSF, multiple care providers were involved. In 44% of the cases with SSF there was a possible or (very) probable relation with perinatal death. International research described a possible or (very) probable relation with perinatal death in 25-30% of all perinatal death cases with substandard care [21]. The Dutch Perinatal Audit has recommended the following: develop uniform care paths, focus on standardised communication and handovers based on the SBAR system (Situation, Background, Assessment, Recommendation), and organise team trainings [22]. It has become clear that within the entire obstetric collaborative network process parameters can be improved. Communication between obstetric care providers within one discipline as well as between different disciplines is important to guarantee an optimal referral process. Moreover, adequate and uniform communication towards the patient (and partner) is important for positive perception [9,18,22].

### Quality of care as perceived by patients

During the last decade, there has been growing interest in quality of care as perceived by patients. With increasing attention to patient-centered care, indicators of care quality more and more involve perceived quality of care and patient satisfaction [23-26]. Measuring patient-reported outcomes is a common strategy used to monitor quality of care in a number of countries. Because of the unique obstetric care system in the Netherlands with different care levels, pregnant women often see different care providers [27]. Recently published data showed that patients who had been referred from primary to secondary care report lower quality of care [28]. These patients received care in more than one institution, from several care providers. Referral during pregnancy and delivery may have a negative effect on a systematic way of communication towards the patients and might cause inconsistency in advice, information, and protocols [28].

### Simulation-based team training

**Figure 2:**
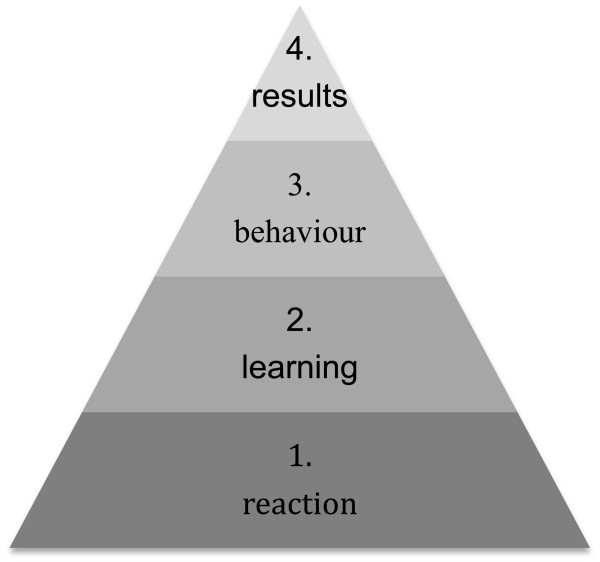
Kirkpatrick’s model for the evaluation of training.

Team training in obstetric emergencies reduces poor perinatal outcome as was shown by a British retrospective cohort study [29]. Recently, a systematic review has concluded that medical simulation is effective for medical education [30]. A meta-analysis showed that simulation-based medical education (SBME) with deliberate practice (DP) is superior in improving medical skills to traditional clinical medical education such as the Halstedian approach (see one, do one, teach one) [31]. DP reflects a life-long period of deliberate effort to improve performance in a specific domain. There are nine elements of DP: 1) high motivation and concentration, 2) well-defined learning objects, 3) appropriate level of difficulty, 4) focused, repetitive practice, 5) rigorous, reliable measurements, 6) feedback, 7) monitoring, error correction, 8) evaluation and performance that may reach a master standard, 9) advancement to the next task [32]. Crew resource management (CRM) has been defined as ‘error management capability to detect, avoid, trap or mitigate the effects of human error and therefore prevent fatal accidents’. It was developed primarily for improving air safety [33]. CRM is a training system that focuses on interpersonal communication, leadership and decision-making. It focuses on the ability of each team member to see, analyse and react, and thereby reducing potential errors. It pursues an open culture where the freedom to respectfully question authority is encouraged. Learning goals of CRM are: enhanced situational awareness, self-awareness, leadership, assertiveness, decision-making, flexibility, adaptability and communication. CRM in team training has shown to improve those competences and results in a positive attitude of trainees towards team building and communication [34]. A positive attitude of trainees, as represented by the first level of Kirkpatrick’s model for evaluation of a training, should result in a learning effect (Kirkpatrick level two) and behavioural change (level three) and finally be translated in patient outcomes (level four) (Figure 2) [35]. A recent study concluded that simulation-based obstetric team training using the concepts of CRM results in a significant improvement in team performance and application of essential medical skills [36]. In our study, multiprofessional teams will be trained in a medical simulation centre with simulated settings that resemble reality as closely as possible. Commercially available high fidelity patient simulators (Noelle™ and Newborn Hal™, Gaumard, Miami, Florida, USA) are used.

## Methods

### Design

The proposed research concerns a transmural multiprofessional simulation-based obstetric team training regarding process management of the Big 4 causes of perinatal mortality. The obstetric collaboration network consisting of ambulance staff, maternity nurses, primary care midwives, obstetric nurses, hospital midwives, residents and obstetricians will be trained.

**Figure 3:**
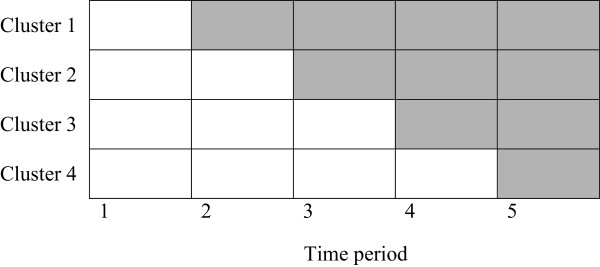
**Stepped wedge design with 4 clusters.** The grey shading indicates the start of the training according the specific cluster.

The Ethical committee did agree that specific ethical approval is not required for this type of study in The Netherlands. Because the study does not interfere with patient care, written informed consent of the participants of the training (care givers) is not indicated. The study is planned to be implemented in a sub-region of the Netherlands (Zuidoost-Brabant) consisting of over one million inhabitants. In this area, around 120 independent midwives are providing primary care. Parallel to this project, in the same region the Regional Consortium Obstetrics Brabant has been founded. This consortium is an association of a perinatology centre (tertiary care: Máxima Medical Centre, Veldhoven), adjacent hospitals (secondary care: Jeroen Bosch hospital Den Bosch, Catharina hospital Eindhoven, Bernhoven hospital Uden, Elkerliek hospital Helmond, St Anna hospital Geldrop) and surrounding primary care midwives. Each hospital with its referring midwifery practices (regional Obstetric Cooperative) is considered to be a separate study group. A stepped wedge trial design will be employed. A stepped wedge trial is a cluster-randomised trial in which all study groups (clusters) receive the intervention by a sequential roll out of the trainings over a number of time periods. Computerised randomisation will define the sequence of the study groups. This design was chosen primarily for logistical reasons and because of the fact that all study groups will eventually receive the team training. Recent literature shows that the stepped wedge trial design has several advantages over a randomised trial, and can offer a number of opportunities. All clusters start in the control condition. The clusters will switch to the intervention at consecutive time points, where the time of the switch is randomised for every cluster. Eventually, all clusters will receive the intervention (Figure 3). The stepped wedge design is useful when the intervention is thought to have a beneficial effect. With a classic cluster trial, randomisation would withdraw the intervention from a part of the study groups. In addition, there are other advantages of the stepped wedge design. First, the clusters act as their own controls because they receive both the control and intervention conditions. Therefore, the intervention effect can be estimated from both between- and within-cluster comparisons. This results in more statistical power and smaller required sample sizes than in a parallel group design. The stepped wedge design is also useful where phased implementation is preferable because of logistical, practical or financial constraints [37-39].

#### Focus groups

Prior to the intervention, focus group interviews were performed. This resulted in insight in topics relevant to patients and care providers, concerning adequate communication and process management. The input of the focus group interviews was used for development of questionnaires for patients and care providers. The focus groups were organised separately in the following categories:

● The pregnant women and women who recently gave birth. These focus group interviews were used to explore what is important to women regarding care during pregnancy and delivery. Based on these focus groups, the Pregnancy and Childbirth Questionnaire (PCQ), to measure quality of care as perceived by women who recently gave birth, has recently been developed and validated [28]. The PCQ will be used for an assessment of all women who recently gave birth before and after the training, concerning the entire Consortium of Brabant.

● The primary care (independent) midwife. The midwives were interviewed to evaluate most common problems in communication. This implies referral of patients to secondary/tertiary care but also aspects of communication when a client is referred back to primary care (during pregnancy/after delivery). The most relevant items were used to construct a questionnaire that will be sent out to all independent midwives for an assessment before and after the training.

● The ‘maternity nurse’. This care provider was interviewed in the focus group together with the primary care midwife. The maternity nurse assists the community midwife during a home delivery. Besides that, after the delivery, she provides care to mother and child at home for about five until eight days. The communication between this nurse, the primary midwife and the patient is very important.

● Secondary care (hospital) midwife. This is an important target group because these midwives are often the first person to contact in case of referral from primary to secondary/tertiary care. Moreover, she is (together with the resident) the first person to contact the obstetrician to inform about the patient.

● The obstetrician and obstetric resident. In case of referral these medical doctors are the finals responsible for the follow-up of pregnancy/delivery. This group was interviewed to evaluate which aspects are crucial in the communication during medical handovers.

### Recruitment and intervention

All obstetric care providers being part of the Consortium Brabant were invited for participating in the study. The Consortium Brabant consists of six hospitals with in total 60 obstetricians. The surrounding primary care consists of approximately 120 primary care midwives organised in about 45 independent midwifery practices. One hospital, St Anna hospital Geldrop with lowest annual delivery rate of around 1.000, decided not to join the team trainings because of logistic reasons. The region of the Máxima Medical Centre was used for a pilot study, leaving four study groups with annual around 9,000 deliveries for this study project. Every hospital with its regional Obstetric Cooperation accounts for one study group. Within the study group, training teams will be formed consisting of ambulance staff (two per team), maternity nurses (one or two per team), primary care midwives (two to five per team), obstetric nurses (two per team), secondary care midwives (one or two per team), residents (one to three per team) and obstetricians (one to three per team), representing the entire obstetric collaborative network with a total of 12–18 care providers per team. There will be two instructors/facilitators per training: one medical instructor (obstetrician) and one communication expert. An expert panel, consisting of representatives of all obstetric care levels, designed obstetric scenarios for the team training, taking into account the topics that have resulted from the focus groups. Training will focus on process management of Big 4 disorders. The focus will mainly be on non-technical skills such as CRM, communication tools and using SBAR, and less on medical technical skills. The team training will take place at the medical education and simulation centre in Eindhoven, the Netherlands (Medsim) [40]. The medical simulation centre pursues a safe learning environment for trainees.

The following four scenarios will be trained:

1. Unexpected home delivery of fetus in breech presentation

2. Extreme preterm delivery starting at home

3. Home delivery with fetal distress and unexpected SGA

4. Unexpected resuscitation of newborn with unexpected congenital heart abnormality at home.

The scenarios are based on national and international guidelines [41-52]. Prior to the training, teams will receive an explanation concerning the equipment and environment. Each trainee will participate actively in at least one scenario and often more. Each scenario will start with an introductory briefing video. Thereafter, the team moves to the simulation room where they manage the simulated patient. State of the art high fidelity patient simulators will be used (Noelle™ and Newborn Hal™, Gaumard, Miami, Florida) and patient actresses. All scenarios will be videotaped (using B-Line Medical® software, Washington, DC). After each scenario a debriefing with reviewing the video recordings will be provided. The instructors will provide feedback on teamwork and skills (medical technical and non-technical) using video recordings. Learning goals based on CRM will be evaluated during the debriefing, such as: attention situational awareness, self-awareness, leadership, assertiveness, decision-making, flexibility, adaptability, and communication tools. There will be a focus on standardised communication and handovers based on the SBAR system (Situation, Background, Assessment, Recommendation).

The nine elements of DB will, if achievable, be applicated to the training by:

1) A syllabus concerning communication tools, CRM and medical knowledge about Big 4 disorders has been written and will be handed out prior to the training. To stimulate the motivation and concentration of the trainee a multiple choice exam prior to the training will be performed.

2) Learning objects:

a. prior to the training, all trainees will be asked to define an individual learning goal

b. learning goals will be defined per specific scenario and will be evaluated during the debriefing

c. take home messages will be hand over to the teams at the end of the training. The teams will work on implementing learning goals in their Obstetric Cooperation.

3) This training will not sufficiently be able to focus on an (individual) appropriate level of difficulty. In general, the scenarios have an increasing difficult level.

4) This training is a one-time training making focused and repetitive practice not achievable. However, to achieve repetitive awareness and use of communication tools based on CRM and SBAR, the study groups will use pocket charts with communication tools in daily practice.

5) Rigorous, reliable measurements: the knowledge of the trainees will be assessed by a multiple-choice exam before and after the training.

6) Feedback will be provided during the debriefing. After each scenario a debriefing session will take place. The debriefing will exist of three phases: reaction of trainees, analysis of performance and take home messages. By reviewing video recordings, feedback will focus on predefined learning goals, on team performance and application of medicals skills.

7) Monitoring and error correction will be performed by reviewing videotaped performance during the debriefing.

8) Evaluation and performance that may reach a master standard: this is not achievable, since there is no definition of what the master standard would be

9) Advancement to the next task: this is not realistic with a one-day training.

After the training, all trainees will fill in an evaluation form about their experiences concerning the training in which they will score (0–5) for 36 different items.

#### In situ simulation

Four months after the intervention, the effect of training on team performance will be measured by so-called unannounced (as far as possible) in situ simulations, during which care providers are assessed on their teamwork within their own working environment. The in situ simulation will consist of one or two scenarios which will be managed by a team consisting of primary and secondary care providers, located at a delivery room in the hospital. The in situ simulation will be videotaped and analysed by independent experts.

### Hypothesis

Multiprofessional simulation-based obstetric team training, using CRM and elements of DP, will improve perinatal outcome, team performance, quality of care as perceived by patients and collaboration of care providers.

Questions to be answered:

1. Does multiprofessional simulation-based obstetric team training improve perinatal outcome?

2. Does multiprofessional simulation-based obstetric team training improve team performance as assessed by an unannounced in situ simulation?

3. Does multiprofessional simulation-based obstetric team training improve quality of care as perceived by patients?

4. Does multiprofessional simulation-based obstetric team training improve collaboration of care providers?

### Outcome measures

Primary outcome will be a composite adverse perinatal outcome as defined by perinatal mortality and/or NICU admission. Data on the primary outcome will be obtained from the Netherlands Perinatal Registry (PRN).

Secondary outcomes will be:

1. 1.Team performance. For measuring team performance, an independent panel of experts will evaluate the videotaped team training sessions and calculate the Clinical Teamwork Scale (CTS) [53].

2. Quality of care as perceived by patients. This will be measured before and after the training by using a questionnaire consisting of the validated PCQ and some additional questions regarding pregnancy, delivery and the first postpartum week [28].

3. Care providers’ satisfaction with teamwork and collaboration between and within the different levels of care. This will be measured before and after the training using a questionnaire which is partly based on the validated Doctors’ Opinions on Collaboration (DOC) questionnaire for general practitioners and medical specialists and adjusted to the obstetric care field [54].

4. Incidence of:

a. Big 4 disorders defined as [18]:

Small for gestational age, defined as a birth weight below the 10^th^ percentile

Preterm delivery before 37 weeks

Congenital anomalies

Five minute Apgar score below 7

b. number of Big 4 pregnancies starting delivery in primary care

c. perinatal mortality

d. fetal mortality rate

e. neonatal mortality rate

f. NICU admission

g. admission to neonatology unit (non-NICU)

h. caesarean section

i. ventouse or forceps delivery

j. episiotomy

k. hemorrhage postpartum (>1000 ml of blood loss)

l. third or fourth degree perineal trauma

### Sample size calculation

In 2010 perinatal mortality rate was 0.9% and the NICU admission rate 2.3%. To avoid double telling, a composite rate of mortality and NICU admission will be around 3%. [5]. The sample size for the study was calculated by using the formula as proposed by Woertman and De Hoop [38]. This formula calculates the design effect required on top of the sample size calculation for a standard randomised clinical trial (RCT). To show a reduction in perinatal mortality and NICU admission rate from 3% to 1.65%, with an alpha of 0.05 and a power of 80%, a total of 4,000 deliveries would be needed for a simple RCT design. The design effect was calculated assuming an intracluster correlation (ICC) of 0.05, a cluster size of 1,800 deliveries per year, and four clusters or study groups. Taking into account the design effect, we need 565 deliveries per measurement period per cluster. To achieve this number we need 16 weeks for each period, adding up to a total study period of 82 weeks including a 16-week control period before the first training. A mixed effects model will be used to model the data and test the hypothesis of no effect from team training to accommodate cluster effects and time effects. Statistical significance will be accepted at a two-sided p-value < 0.05. In the study region 9,000 deliveries occur per year, with a minimum of around 1,806 deliveries per year per study group (cluster) and a maximum of around 3,500.

## Discussion

As far as we know now, transmural multiprofessional simulation-based obstetric team training, using CRM and elements of DP, integrating the entire obstetric collaborative network, has never been studied before. We hypothesise that this obstetric team training improves perinatal outcome, team performance, quality of care as perceived by patients, and collaboration between care providers. The current project fits well within one of the main goals of the Dutch government to set up research that can prevent avoidable perinatal mortality and morbidity. Management of obstetric scenarios, based on the Big 4 causes of perinatal mortality, will be practiced in a medical simulation centre by teams with representatives of the obstetric collaborative network. The innovative aspect of the current project is the focus on non-technical skills (CRM, SBAR) rather than technical skills with the application of the elements of DP and defining learning goals based on CRM and the fact that different care providers will be trained together in one integrative cooperating team. Because team training and communication training has shown to be effective in secondary obstetric care [36], there is no reason to believe that this will not work within team training with integrating care providers from primary, secondary and tertiary care. To achieve a better ranking position concerning perinatal mortality rates in Europe, it is necessary to intensify an integrative organisation of obstetric care in the Netherlands in which all different care levels will integrate, in which uniform care paths will be developed forming ‘patient centered care’. This is in line with the recent letter of the Minister of Health which has been sent to the House of Parliament in which she focuses on the development of an integrative obstetric health system.

## Abbreviations

CRM: Crew resource management; CTS: Clinical teamwork scale; DOC: Doctors’ opinion on collaboration; DP: Deliberate practice; Medsim: Medical education and simulation centre Eindhoven; NICU: Neonatal Intensive Care Unit; OHC: Obstetric high care; PRN: National Dutch Perinatal Registry; SBAR: Situation, Background, Assessment, Recommendation; SBME: Simulation-Based Medical Education; SGA: Small for gestational age; SSF: Substandard factors; ZonMw: the Netherlands Organization for Health, Research and Development.

## Competing interests

The authors declare that they have no competing interests.

## Authors’ contributions

All authors were involved in conception and design of the study. All authors participated in the design of the study during several meetings and are local investigators at the participating centers. All authors drafted the manuscript. All authors edited the manuscript and read and approved the final manuscript.

## Pre-publication history

The pre-publication history for this paper can be accessed here:

http://www.biomedcentral.com/1472-6920/14/175/prepub
